# Research Progress on the Influence of Novel Targeted Drugs for Osteoporosis on Glucose Metabolism

**DOI:** 10.3390/biom15030331

**Published:** 2025-02-25

**Authors:** Lingyang Meng, Lei Sun, Mei Li

**Affiliations:** Key Laboratory of Endocrinology of National Ministry of Health, Department of Endocrinology, Peking Union Medical College Hospital, Chinese Academy of Medical Science and Peking Union Medical College, Beijing 100730, China; lyangm987@student.pumc.edu.cn (L.M.);

**Keywords:** glucose metabolism, osteoporosis, diabetes, denosumab, sclerostin, Dickkopf-1

## Abstract

Both diabetes and osteoporosis are serious chronic conditions. Evidence is mounting that several bone-derived hormones play a role in glucose metabolism in patients with diabetes. Notably, novel biotargeted anti-osteoporotic agents have been recently found to reduce the risk of diabetes. This review explores the correlation of osteokines, including the receptor activator of nuclear factor-κB ligand (RANKL), sclerostin, and Dickkopf-1 (DKK1) with glycemic indicators in patients with diabetes, as well as the effects of their respective monoclonal antibodies on glucose metabolism and their possible mechanisms. Denosumab, the monoclonal antibody against RANKL, has been shown to reduce glycated hemoglobin (HbA1c) and the risk of diabetes, possibly by enhancing pancreatic β-cell survival and glucagon-like peptide-1 secretion. Sclerostin was positively correlated with HbA1c and may induce insulin resistance via endoplasmic reticulum stress. The association of DKK1 with fasting plasma glucose and HbA1c is still unclear, though decreasing DKK1 levels may correlate with β-cell survival. However, few studies have investigated the effects of antibodies against sclerostin or DKK1 on glucose metabolism. Further research is required to elucidate the influence of novel anti-osteoporotic biotargeted agents on glucose homeostasis in patients with diabetes and their underlying mechanisms.

## 1. Introduction

Diabetes mellitus (DM), a chronic disease characterized by hyperglycemia associated with abnormalities of insulin secretion and/or utilization [1], represents a global public health concern that poses a serious threat to human well-being. In 2021, there were 529 million patients with diabetes worldwide [2]. By 2050, this number is projected to exceed 1.3 billion [2].

Recently, growing evidence has indicated that bone plays a vital role in metabolic processes through the secretion of several osteokines [3,4]. Some of them have been experimentally demonstrated to be beneficial to glucose metabolism—for instance, osteocalcin, which enhances glucose homeostasis by promoting pancreatic islet β-cell proliferation [5], insulin secretion [6], and insulin sensitivity of liver, skeletal muscle, and white adipose tissue [6,7,8]. Consistently, lower circulating osteocalcin levels are associated with an increased risk of diabetes [9]. Similarly, osteoblast-derived lipocalin 2 has been demonstrated to stimulate insulin secretion of β-cell, improve insulin sensitivity and glucose tolerance, and suppress appetite thereby reducing food intake in mice [10]. By contrast, other bone-derived hormones exert detrimental effects on glucose metabolism. The receptor activator of nuclear factor-κB (NF-κB) ligand (RANKL) secreted by osteoblasts induces hepatic insulin resistance by activating NF-κB signaling, and β-cell dysfunction via the p38 mitogen-activated protein kinase (MAPK) signaling pathway [11]. Similarly, sclerostin, secreted by osteocytes, is highly secreted in patients with diabetes [12,13] and leads to compromised glucose tolerance and insulin sensitivity [14,15].

With the prominent physiological functions in bone metabolism, some osteokines are biologically targeted for the treatment of osteoporosis, and the application of these targeted drugs has yielded promising effects on improving glucose metabolism in patients with osteoporosis. The monoclonal anti-RANKL antibody, denosumab, exhibited benefits in decreasing glycated hemoglobin (HbA1c) in osteoporotic patients [16,17,18]. Notably, denosumab was recently reported to significantly reduce the risk of diabetes [19,20,21], but the underlying mechanisms have not been fully understood. In addition, studies are insufficient on whether antibodies against sclerostin or Dickkopf-1 (DKK1), the inhibitors of the Wnt/β-catenin pathway, promote glucose metabolism. This review will summarize the effects of novel biotargeting agents for osteoporosis on glucose homeostasis as well as the potential mechanisms and envision the therapeutic strategies for osteoporosis combined with diabetes.

## 2. Effects of Denosumab on Glucose Metabolism

Denosumab, the humanized monoclonal antibody against RANKL, is the first biological therapy for osteoporosis by inhibiting the osteoprotegerin (OPG)/RANKL/RANK pathway, which plays a pivotal role in bone metabolism via stimulating osteoclastogenesis and thereby promoting bone resorption [22,23]. By targeting against RANKL, denosumab exerts a potent antiresorptive effect through uncoupling the interaction between osteoblasts and osteoclasts and reducing bone resorption, which yields a remarkable increase in bone mineral density (BMD) in osteoporotic patients [23,24].

With the discovery of the negative impact of RANKL on glucose metabolism [25,26,27,28,29,30], mechanistic and clinical studies elaborating on the effects of denosumab on plasma glucose and diabetes development are on the rise, although the evidence is not quite conclusive.

### 2.1. The Mechanism of Denosumab Affecting Glucose Metabolism

Recently, studies reported that the OPG/RANKL/RANK pathway plays an adverse role in maintaining glucose homeostasis [11], as RANKL induces insulin resistance [25,26] as well as the apoptosis and impairment of β-cell [27,28,29,30].

**Figure 1:**
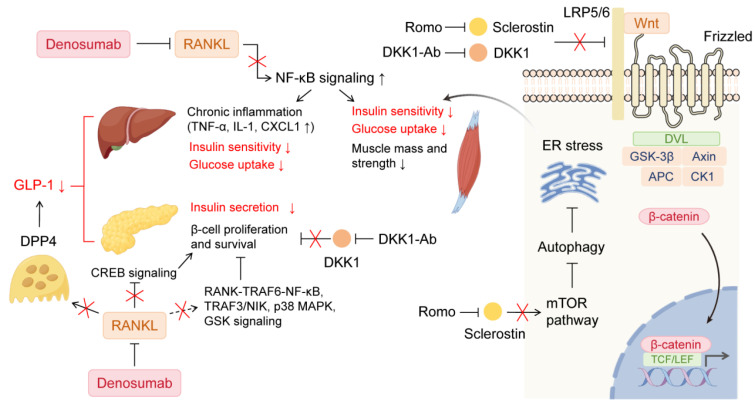
The influence of RANKL, sclerostin, and DKK1 on glucose metabolism and the corresponding signaling cascade. RANKL exerts detrimental effects on glucose homeostasis through multiple pathways. By activating NF-κB signaling pathway, RANKL induces high level of TNF-α, IL-1 and CXCL1 expression, causing hepatic chronic inflammation, and reduces insulin sensitivity, glucose uptake, and possibly skeletal muscle mass and strength. Additionally, RANKL impairs β-cell survival and proliferation, potentially via activating RANK-TRAF6-induced NF-κB, TRAF3/NIK, p38 MAPK, and GSK pathways and by inhibiting CREB signaling. Additionally, RANKL stimulates DPP4 secretion from osteoclasts, which degrades GLP-1, leading to insulin resistance and decreased glucose uptake in the liver and skeletal muscle, and lower insulin secretion. Denosumab may mitigate the negative effects mentioned above by targeting RANKL. Both sclerostin and DKK1 inhibit the binding of Wnt and LRP5/6, thereby reducing the stability of β-catenin, which transfers into the nucleus and promotes expression of downstream genes. Sclerostin could induce skeletal muscle insulin resistance via upregulation of ER stress by mTOR-mediated suppression of autophagy. DKK1 is speculated to have a negative relationship with β-cell proliferation and survival. Romosuzumab and DKK1-Ab may improve glucose metabolism by inhibiting these processes, respectively. CREB, cyclic-AMP response binding protein; CXCL1, chemokine (C-X-C motif) ligand 1; DKK1, Dickkopf-1; DPP4, dipeptidyl peptidase-4; ER, endoplasmic reticulum; GLP-1, glucagon-like peptide-1; GSK, glycogen synthase kinase; IL-1, interleukin-1; LRP5/6, lipoprotein receptor-related protein 5/6; MAPK, mitogen-activated protein kinase; mTOR, mammalian target of rapamycin; NIK, NF-κB-inducing kinase; NF-κB, nuclear factor kappa B; RANKL, receptor activator of nuclear factor kappa B ligand; Romo, romosuzumab; TNF-α, tumor necrosis factor α; TRAF, tumor necrosis factor receptor-associated factor. Dashed lines indicate the presumed mechanism.

RANKL inhibition has witnessed an improvement in glucose metabolism in both in vitro and in vivo studies (Figure 1). Systemic or hepatic RANKL knockout in mice improved hepatic insulin sensitivity and glucose tolerance [25]. RANKL inhibition through OPG, denosumab, or other RANKL inhibitors not only increased glucose uptake and attenuated subacute inflammation and insulin resistance in skeletal muscle [26] and liver [25] but also enhanced human β-cell proliferation. RANKL overexpression mice (HuRANKLTg+) exhibited impaired insulin tolerance and lower glucose uptake in muscle compared to the wild type, which was restored through OPG or denosumab treatment [26]. Both agents also reversed the mRNA levels of NF-κB pathway-related genes in chronic RANKL or insulin-treated C2C12 myotubes, and OPG reduced glucose levels in their supernatants [26]. Kondegowda et al. found that OPG induced activation of cyclic-AMP response binding protein and inactivation of glycogen synthase kinase (GSK) by increasing their phosphorylation, both of which promoted rodent and human β-cell replication [31]. They also demonstrated that denosumab enhanced human β-cell proliferation as a significantly increased percentage of Ki67-positive or BrdU-positive cells in human islet cell cultures or human islet grafts in the kidney capsule of non-obese diabetic/severe combined immunodeficient mice treated with denosumab was observed, respectively [31]. An in vitro study showed that tumor necrosis factor receptor-associated factor 3 (TRAF3)/NF-κB-inducing kinase (NIK) mediated RINm5F cells apoptosis induced by high glucose, which was reversed by sRANKL-IN-3, a RANKL inhibitor, combined with interference of TRAF3 expression [32]. Given that RANKL induces human β-cell apoptosis and β-cell mass impairment via activating p38 MAPK and non-canonical NF-κB signaling [11], denosumab may also stimulate β-cell proliferation through both pathways. More importantly, denosumab-treated postmenopausal nondiabetic women had a significant decrease in serum dipeptidyl peptidase-4 (DPP4) and an increase in glucagon-like peptide-1 (GLP-1) levels, the former identified as an osteoclast-derived factor as its mRNA expression was overt in osteoclasts, but not in other bone cell types [18]. Since DPP4 inhibitors lower plasma glucose by increasing GLP-1 levels [33], this is regarded as a potential mechanism by which denosumab improves glucose metabolism. However, how denosumab inhibits DPP4 secretion from osteoclasts is not elucidated and warrants further investigation.

Besides, denosumab has also been shown to protect β-cells from damage in type 1 DM (T1DM). Kondegowda et al. found that denosumab prevented human primary β-cells from proinflammatory cytokines-induced cell death by blocking the interaction of RANK with TRAF6, which is critical for NF-κB activation [34].

Denosumab has been found to affect the levels of osteocalcin, a bone-derived hormone that improves glucose metabolism by enhancing insulin secretion and human β-cell proliferation [35,36]. Higher osteocalcin level was associated with a reduced risk of diabetes in a cohort of 5396 participants without diabetes or osteoporosis during a mean follow-up of 4.6 years [9]. Paradoxically, a randomized clinical trial (RCT) revealed that a 2-year denosumab treatment dramatically decreased osteocalcin levels in postmenopausal women with osteoporosis [37]. Similarly, a prospective cohort study also found a reduction of osteocalcin levels in postmenopausal osteoporotic women after a 24-week denosumab treatment [38]. Taken together, the antidiabetic effect of denosumab was presumably not mediated by osteocalcin, which remains to be clarified.

### 2.2. Insulin Secretion and Insulin Resistance

Most studies indicated that in nondiabetic postmenopausal women with osteoporosis, denosumab did not exert significant effects on insulin secretion or insulin resistance [16,18,39]. A prospective cohort study involving 48 postmenopausal osteoporotic nondiabetic women showed that 1-month denosumab therapy decreased fasting insulin by 10.5 pmol/L and homeostatic model assessment of insulin resistance (HOMA-IR) by 0.5 (Table 1) [38]. However, 3- or 6-month denosumab treatment did not bring significant changes in fasting insulin or HOMA-IR compared with that of baseline [38]. No plausible explanation for this transient variation was given, making it unreliable due to the small sample size.

Few studies have focused on the effect of denosumab on insulin resistance in osteoporotic patients with diabetes. A prospective cohort study that included 20 osteoporotic patients with type 2 DM (T2DM) found that 12 months of denosumab therapy resulted in a decrease of 0.6 in HOMA-IR from baseline, but there was no change in HOMA-IR at 6 months [17]. Since they did not exclude the effects of antidiabetic medication (ADM) (other than insulin) and the sample size was small, whether denosumab improves insulin secretion and resistance warrants further validation in a large RCT or cohort study.

### 2.3. Fasting Plasma Glucose (FPG)

For postmenopausal women with or without diabetes, FPG does not seem to be affected by denosumab administration [16,17,18,38,39,40,41,42]. Post hoc analyses of the FREEDOM trial, a 3-year placebo-controlled trial showing that denosumab reduced the risk of vertebral, nonvertebral, and hip fractures in postmenopausal women with osteoporosis, revealed that denosumab therapy for 3 years did not affect FPG in participants with normal glucose tolerance [40], prediabetes [42], or diabetes with ADM [42] compared with placebo group, while for those with diabetes not using ADM, a 3-year denosumab therapy decreased the FPG by 6.8mg/dL [42]. The reason may be that the ADM masked the effect of denosumab on glucose levels. In an RCT including 46 postmenopausal nondiabetic women, FPG did not change significantly with denosumab treatment for 3 months [18]. Similarly, three prospective cohort studies showed no changes in FPG in postmenopausal nondiabetic women receiving denosumab for 1 to 6 months [16,38,39]. Additionally, for premenopausal nondiabetic women with breast cancer receiving ovarian function suppression and aromatase inhibitor (AI), no effects were detected in FPG after 12 months of denosumab therapy [41]. Taken together, denosumab seems to have little influence on glucose homeostasis in women with normal glucose tolerance. Since denosumab has been shown to promote GLP-1 secretion, it may be explained by the glucose-dependent mechanism by which GLP-1 stimulates insulin secretion [18]. In a case-control study including 345 osteoporotic patients with T2DM or prediabetes, 6 months of denosumab treatment achieved a ~13% reduction in FPG compared with oral or intravenous bisphosphonates, but there was no difference at 12 months of treatment [18]. Nonetheless, given no blinding and exclusion of confounders in this study, the results should be interpreted with caution.

### 2.4. HbA1c

Denosumab appeared to exhibit a favorable effect on HbA1c in osteoporotic patients with T2DM or prediabetes. In the case–control study above [18], compared to bisphosphonates, 6 and 12 months of denosumab therapy yielded approximate 0.4% and 0.6% decreases in HbA1c, respectively. A prospective observational study including 20 patients with osteoporosis and T2DM also reported a 0.2% reduction of HbA1c with 12-month denosumab treatment compared with baseline [17]. However, the results of the two studies were not robust since they did not exclude the interference of ADM.

For postmenopausal osteoporotic women without diabetes, the results of HbA1c were contradictory. An RCT that recruited 68 premenopausal nondiabetic women with breast cancer receiving ovarian function suppression and AI did not observe significant changes in HbA1c after 3, 6, or 12 months of denosumab therapy [41]. A prospective cohort study found a 3.6 mmol/mol increase in HbA1c following 5 months of denosumab treatment on 14 postmenopausal osteoporotic nondiabetic women with breast cancer treated with AI [39]. Since the potential negative effects of menopause on glucose metabolism [43], compared to the premenopausal, postmenopausal women may be more susceptible to AI, which was reported to disrupt glucose homeostasis in breast cancer patients by interfering with the estrogen-insulin interplay [44]. This may contribute to the discordant results between the two studies apart from the different sample sizes, denosumab treatment duration, and study design. Conversely, another prospective cohort study including 14 postmenopausal osteoporotic women without diabetes showed a decrease of 1.2 mmol/mol in HbA1c after treatment with denosumab for 3 months [16]. This result needs to be confirmed due to the small sample size, short duration of treatment, and uncertainty regarding whether dietary control or exercise was applied.

**Table 1:** Clinical studies on the effects of denosumab on glucose metabolism.

Study Design	Subjects	Targeted Therapy Group	Follow-Up (months)	FPG	HbA1c	Insulin Secretion	Insulin Resistance	Risk of Incident Diabetes	Reference
Real world study	84,624 nondiabetic osteoporotic patients	42,312	60	NA	NA	NA	NA	↓ 17%	[45]
Retrospective cohort study	68,510 nondiabetic osteoporotic patients	34,255	22.8	NA	NA	NA	NA	↓ 16%	[20]
Real world study	25,339 nondiabetic osteoporotic patients	4301	26.4	NA	NA	NA	NA	↓ 32%	[19]
New-user comparative study	30,422 nondiabetic osteoporotic patients	3354	60	NA	NA	NA	NA	↓ 27%	[21]
Randomized clinical trial	68 premenopausal nondiabetic women with breast cancer treated with ovarian function suppression and AI	34	0, 3, 6, 12	—	—	NA	NA	NA	[41]
Randomized clinical trial	46 postmenopausal nondiabetic women	22	3	—	NA	—	—	NA	[18]
Case-control study	345 osteoporotic patients with T2DM or prediabetes	115	6	↓ ~13%	↓ ~0.4%	NA	NA	NA	[18]
			12	—	↓ ~0.6%	NA	NA	NA	
Post hoc analysis of clinical trial	7076 postmenopausal osteoporotic nondiabetic women	3535	36	—	NA	NA	NA	—	[40]
Post hoc analysis of clinical trial	666 postmenopausal osteoporotic women with diabetes receiving ADM	342	36	—	NA	NA	NA	NA	[42]
	294 postmenopausal osteoporotic women with diabetes without ADM	151	36	↓ 6.8 mg/dL	NA	NA	NA	NA	
	1268 postmenopausal osteoporotic women with prediabetes	628	36	—	NA	NA	NA	NA	
Prospective cohort study	48 postmenopausal osteoporotic nondiabetic women	48	1	—	NA	↓ 10.5 pmol/L	↓ 0.5 (HOMA-IR)	NA	[38]
			3, 6	—	NA	—	—	NA	
Prospective cohort study	14 postmenopausal osteoporotic nondiabetic women	14	1	—	—	—	—	NA	[16]
			3	—	↓ 1.2 mmol/mol	—	—	NA	
Prospective cohort study	14 postmenopausal osteoporotic nondiabetic women with breast cancer treated with AI	14	1	—	NA	—	—	NA	[39]
			5	NA	↑ 3.6 mmol/mol	NA	NA	NA	
Prospective cohort study	20 osteoporotic patients with T2DM	20	6	—	—	NA	—	NA	[17]
			12	—	↓ 0.2%	NA	↓ 0.6 (HOMA-IR)	NA	

↑ significantly increased; ↓ significantly decreased; — no significant changes; ADM, antidiabetic medication; AI, aromatase inhibitor; FPG, fasting plasma glucose; HbA1c, glycated hemoglobin A1c; HOMA-IR, homeostatic model assessment of insulin resistance; NA, not applicable; T2DM, type 2 diabetes mellitus.

### 2.5. Denosumab and the Risk of Diabetes

Recently, several large-scale real-world studies demonstrated that denosumab could reduce incident diabetes in osteoporotic patients (Table 1) [19,20,21]. In a study involving 68,510 nondiabetic osteoporotic patients, continuous denosumab treatment for 1.9 years was associated with a 16% reduction in diabetes risk (hazard ratio [HR] 0.84, 95% confidence interval [CI] 0.78–0.90) compared with discontinued denosumab treatment [20]. This was only observed in adults aged 65 years or older, regardless of cardiovascular or renal comorbidities [20]. Another study enrolled 25,339 nondiabetic osteoporotic patients aged 45 years or older and found that switching to denosumab from oral bisphosphonate or initiating denosumab for 2.2 years was associated with a 32% decreased risk (HR 0.68, 95% CI 0.52–0.89) of T2DM compared with patients receiving a continued oral bisphosphonate [19]. Moreover, patients with a high risk of T2DM, such as prediabetes or obesity, obtained a further decreased diabetes risk with denosumab therapy compared with those using oral bisphosphonate [19]. Consistently, another new-user study on 30,422 nondiabetic osteoporotic patients aged 45 years or older presented that 5-year denosumab administration was associated with a 27% decreased risk (HR 0.73, 95% CI 0.58–0.89) of developing T2DM compared with alendronate therapy [21]. However, the FREEDOM trial did not observe a significant effect of denosumab on reducing incident diabetes among postmenopausal women [40]. An explanation may be that the proportion of overweight or obesity in this trial was lower than that in the general population of comparable age [40], resulting in a lower risk of diabetes in these subjects. A recent real-world cohort study involving osteoporotic patients without diabetes compared 42,312 participants treated with denosumab and the same number of those receiving bisphosphonates for 5 years. The study found that denosumab significantly reduced the risk of incident T2DM (HR 0.83, 95% CI 0.78–0.88), particularly in older patients and those who were overweight or obese [45]. This study further analyzed long-term outcomes in osteoporotic patients with T2DM prescribed denosumab or bisphosphonates (*n* = 5752 for each). Denosumab brought a 21% decreased all-cause mortality (HR 0.79, 95% CI 0.72–0.87) (log-rank *p* = 0.05) and a 33% reduced risk of foot ulceration (HR 0.67, 95% CI 0.53–0.86) (log-rank *p* = 0.01). Moreover, a meta-analysis of 4 research (3 cohort studies and the FREEDOM trial) showed that denosumab was associated with a reduced risk of developing T2DM (relative risk [RR] 0.83, 95% CI 0.79–0.87) [45]. The clinical evidence above suggests an association between long-term denosumab therapy and a reduced risk of incident T2DM in patients with osteoporosis without diabetes, particularly in older patients and those with overweight or obesity and demonstrates benefits in improving long-term prognosis and reducing the risk of foot ulcers in patients with osteoporosis and T2DM. However, since most of these studies were observational, they could not draw causation and did not rule out confounding factors such as exercise, diet, and socioeconomic status. Therefore, well-designed RCTs for patients with both osteoporosis and diabetes are needed to validate these benefits of denosumab [46].

Although denosumab improves β-cell survival in vitro, as mentioned above, to date there is no clinical evidence that denosumab reduces the risk of T1DM.

## 3. Effects of Anti-Wnt Inhibitors Antibodies on Glucose Metabolism

Wnt signaling acts as a crucial regulator of bone development and bone mass homeostasis, and Wnt activation promotes bone formation and bone mass [47]. Wnt signals also induce mesenchymal multipotent progenitor cells to differentiate towards osteoblasts.

Two Wnt signaling inhibitors, sclerostin, and DKK1, prevent the combination between Wnt and cell surface receptors—including lipoprotein receptor-related protein 5/6 (LRP5/6) and Frizzled protein—thus indirectly enhancing bone resorption (Figure 1) [48]. Hence, monoclonal antibodies against both molecules respectively, as well as dual antibodies, have been developed as novel biotargeted agents for osteoporosis treatment [49,50]. Since sclerostin and DKK1 levels are significantly elevated in diabetes [12,13,51,52,53,54,55,56] and Wnt pathways also participate in glucose metabolism and T2DM development [57], recent studies have begun to focus on whether these antibodies also exert an effect on glucose homeostasis.

### 3.1. Anti-Sclerostin Antibodies

Sclerostin is a glycoprotein secreted by osteocytes that binds to the extracellular region of LRP5/6 co-receptors, antagonizing the canonical Wnt/β-catenin pathway and inhibiting bone formation by hindering osteoblast proliferation and differentiation [58,59]. This effect can also be achieved via sclerostin competitively binding to the transmembrane serine–threonine kinase receptor of bone morphogenetic protein [60]. Additionally, sclerostin increases the expression of RANKL by osteoblasts and promotes bone resorption [61].

Previous studies have demonstrated that sclerostin levels are elevated in individuals with glycolipid metabolism disorders. A cross-sectional study found that sclerostin levels gradually increased from healthy controls to prediabetes and diabetes patients [53]. In T2DM, there is a gender difference in that males tend to have higher levels of sclerostin than females [12,52,62], which is independent of age [52]. Additionally, the bone tissue of patients with T2DM showed remarkably higher sclerostin mRNA levels than that of nondiabetic subjects [63,64]. Obese individuals with insulin resistance presented significantly higher sclerostin mRNA expression in blood compared with those without insulin resistance [65].

#### 3.1.1. Potential Mechanisms of Sclerostin Affecting Glucose Metabolism

Very few studies investigated how sclerostin influences glucose metabolism. A mechanistic study revealed that sclerostin knockout mice exhibited increased insulin-stimulated glucose uptake in skeletal muscle and improved insulin sensitivity [14]. In contrast, serum insulin levels were significantly elevated in sclerostin-overexpression mice, indicating the occurrence of insulin resistance [14]. Similarly, sclerostin treatment aggravated insulin signaling (e.g., reduced phosphorylation of insulin receptor substrate 1) in C2C12 myocytes and skeletal muscle and liver of mice, causing impaired glucose tolerance and insulin sensitivity in mice, which was alleviated by interference of sclerostin expression [15]. At the molecular level, sclerostin treatment increased the expression of endoplasmic reticulum (ER) stress markers and mammalian target of rapamycin (mTOR) phosphorylation while reducing autophagy markers, which was restored by sclerostin suppression [15]. Although this study proposed that ER stress caused by mTOR-mediated suppression of autophagy contributes to sclerostin-induced glucose metabolism impairment, the causation among them was not rigorously verified. Besides, they did not demonstrate if mTOR inhibition would attenuate sclerostin-induced insulin signaling impairment. Further studies are needed to elucidate whether romosuzumab or sclerostin aptamers function through the mTOR signaling or other pathways to improve glucose metabolism.

#### 3.1.2. Sclerostin and Glucose Metabolism Indicators

Glycemic parameters correlate differently with sclerostin levels in populations with distinct glucose statuses.

• Insulin secretion and insulin resistance

In normoglycemic adults, no significant association was observed between sclerostin levels and insulin resistance or insulin secretion [66,67,68]. Using multiple linear regression analyses, Yu et al. identified a significantly negative correlation between sclerostin levels and fasting insulin levels or HOMA-IR in a longitudinal cohort of 1778 adults without a history of T2DM during a mean follow-up of 7.5 years [67]. A cross-sectional study of prediabetes recruited 79 individuals with impaired glucose regulation and found that HOMA-IR as well as the hepatic and adipose tissue insulin resistance indexes were positively correlated with sclerostin levels, indicating that sclerostin may play a role in the response to insulin of peripheral tissues [69].

• FPG

Yu et al.’s study reported that serum sclerostin levels were not associated with FPG [67], whereas another cohort of 20 normoglycemic individuals revealed an inverse association [66]. The difference can be explained by the disparity in sample sizes and the fact that the former was adjusted for covariates while the latter was not. For patients with prediabetes [69] or T2DM [52], this association was found to be positive. In a meta-analysis, high sclerostin levels were associated with a higher risk of elevated FPG [70]. FPG was also positively correlated with sclerostin mRNA levels in bone tissues of postmenopausal women with T2DM [64]. However, a cross-sectional study of 40 patients with T2DM did not find this association [13], possibly owing to the internal heterogeneity in years of diagnosis (1–26 years). In children and adolescents with T1DM, no association between circulating sclerostin levels and FPG was identified [13].

• HbA1c

HbA1c was presented as positively correlated with sclerostin levels in prediabetes and T2DM [52,69]. An inverse correlation between sclerostin levels and HbA1c was found in children and adolescents with T1DM, which could be explained by the negative association between sclerostin and leptin, the latter being reported to enhance insulin secretion and normalize HbA1c levels [55].

• The risk of diabetes

Whether or not sclerostin levels correlate with the risk of T2DM remains controversial, as two meta-analyses drew opposite conclusions [70,71]. A genome-wide association meta-analysis included 33,961 European individuals from 9 studies [71]. They found that genetically predicted lower sclerostin increased the risk of T2DM (odds ratio (OR) 1.32, 95% CI 1.03, 1.69) [71]. Conversely, another meta-analysis analyzed 5069 participants from two large cohorts (LURIC and ALSPAC mothers), and presented that high sclerostin levels were associated with a higher risk of diabetes, which was deemed as a risk factor for cardiovascular disease [70]. The conflicting findings may be attributed to the following reasons. Firstly, the former study used Mendelian randomization to predict the causal effects of sclerostin and T2DM risk, while the latter was a cross-sectional study that was vulnerable to reverse causation, and it did not rule out the confounding effects of medication. Secondly, the latter study had great internal heterogeneity in the prevalence of diabetes (40.3% in LURIC, 1.8% in ALSPAC mothers). Since the former study incorporated both cohorts analyzed in the latter, it encompassed more diverse populations across varying health conditions, thereby mitigating selection bias. Overall, the effects of sclerostin on diabetes warrants further validation in RCTs applying sclerostin inhibitors, which will be discussed later.

Additionally, Yu et al.’s cohort mentioned above did not identify a significant correlation between sclerostin levels and the risk of T2DM [67]. In this study, the sclerostin levels were not measured during the follow-up, and the self-reported diagnosis of diabetes had relatively low sensitivity (77.8%), both of which may lead to the underestimation of diabetes incidence.

#### 3.1.3. Effects of Anti-Sclerostin Antibodies on Glucose Metabolism

Romosozumab, the human immunoglobulin G2 monoclonal antibody that blocks the action of sclerostin, promotes bone formation and decreases bone resorption, thereby increasing BMD and reducing fracture risk in osteoporotic patients [47].

The effects of romosozumab on glucose metabolism are not well studied. High-fat diet (HFD) mice receiving sclerostin-neutralizing antibodies exhibited a decrease in random-fed glucose and insulin levels, which was likely attributed to the improvement in glucose and insulin tolerance [14]. No clinical studies have reported how glucose metabolism would change following romosozumab therapy in osteoporotic patients. To avoid the cardiovascular and cerebrovascular risk of romosozumab reported by clinical trials [72,73], Apc001PE, a nucleic acid aptamer specifically targeting the loop3 of the sclerostin molecule has been developed [74]. However, whether this aptamer influences plasma glucose or insulin secretion and sensitivity is unclear. It would be worthwhile to investigate the roles of loop3 in glucose metabolism, which might endow the aptamer with a new function in promoting glucose metabolism in osteoporosis patients [59].

### 3.2. Anti-DKK1 Antibodies

DKK1, expressed by mature osteoblasts and osteocytes, binds to the transmembrane channel receptor protein Kremen of LRP5/6 and causes the internalization and degradation of LRP5/6 from the cell surface, reducing the stability of β-catenin and inhibiting bone formation [48,50].

Few studies reported an association between DKK1 levels and glycemic parameters. Circulating DKK1 levels are higher in patients with diabetes and obese patients with insulin resistance compared with healthy controls [56,65,75,76,77]. DKK1 mRNA levels in bone tissues of patients with T2DM were not different from that of nondiabetic control [64]. Patients with T2DM treated with ADM witnessed a significant reduction in DKK1 levels [75,78].

#### 3.2.1. Potential Mechanisms and Influence of DKK1 on Glucose Metabolism

Anti-DKK1 antibody shows promise in osteoporosis treatment, as ovariectomized mice and rhesus macaques displayed a significant increase in BMD after receiving an anti-DKK1 neutralizing antibody [50]. Moreover, a bispecific antibody against DKK1 and sclerostin (hetero-DS) was developed and exhibited greater bone formation and repair efficacy in fracture rat models [79].

However, no study so far reported DKK1’s role in glucose homeostasis in patients with osteoporosis. Studies on the effects of anti-DKK1 antibody or hetero-DS on glucose metabolism are lacking. Presumably, anti-DKK1 antibody reduced circulating DKK1 levels and attenuated diabetic cardiac damage by inhibiting G protein-coupled receptors dysregulation due to membrane LRP5/6 endocytosis in high-fat/high-fructose diet mice [80]. Additionally, increased DKK1 mRNA expression was identified in nonregenerative human bone marrow (BM)-derived multipotent stromal cells (MSCs) compared with regenerative BM-MSCs, the latter’s conditioned culture media significantly increasing the number of total and live human β-cells, which indicated that activated Wnt signaling may promote β-cell survival and proliferation [81]. Collectively, the roles of DKK1 in glucose metabolism and whether DKK1 inhibition yields a benefit on glycemic control in osteoporosis merits further investigation.

#### 3.2.2. DKK1 and Glucose Metabolism Indicators

• Insulin secretion and insulin resistance

A cross-sectional study indicated that no correlation existed between DKK1 levels and fasting insulin or HOMA-IR among nondiabetic individuals [82]. A positive association of DKK1 levels with average total insulin dose was identified in children and adolescents with T1DM [77], indicating that DKK1 levels may reflect the extent of insulin deficiency in T1DM.

• FPG

There is no consistent conclusion on the relationship between DKK1 levels and FPG. A cross-sectional study revealed a positive correlation between DKK1 levels and FPG in 40 children and adolescents with T1DM [77]. In a small RCT of 20 patients with T2DM randomized into acarbose or placebo treatment, both DKK1 levels and FPG decreased significantly in the 20-week acarbose group, and a significant correlation was observed between the changes in DKK1 and FPG [75]. However, a cross-sectional study indicated that no correlation existed between serum DKK1 levels and FPG among 121 nondiabetic men older than 40 years [82]. The DKK1 changes were not associated with the improvement of FPG in post hoc analysis of an RCT involving 92 patients with T2DM, who were assigned to acarbose or metformin treatment for 12 weeks [78]. Given the different glycemic status of the participants, sample sizes, duration of ADM, and no control of confounders among these studies, the interaction between DKK1 and hyperglycemia should be interpreted cautiously.

• HbA1c

Subjects with lower baseline DKK1 had significantly lower baseline HbA1c levels in a post hoc analysis of a clinical trial that compared the effect of 12-week acarbose and metformin treatment on glycemic variability in patients with T2DM receiving premixed insulin, but the DKK1 changes were not associated with the improvement of HbA1c [78]. Since the lack of a control group and no adjustment for nutrition intake, the findings cannot be generalized. The association of DKK1 levels with HbA1c was not observed in children and adolescents with T1DM [77]. Large cohorts are needed to clarify the correlation between DKK1 levels and long-term glycemic control.

### 3.3. The Crosstalk Between Wnt/β-catenin Signaling and Glucose Metabolism

The relationship between Wnt/β-catenin signaling and glucose metabolism is complicated. Activation or inhibition of Wnt downstream signaling have both been found to enhance glucose metabolism [57]. Transcription Factor 7 Like 2 (TCF7L2) is a downstream effector molecule of the Wnt pathway. HFD-fed liver-specific *Tcf7l2* knockdown mice were found to suffer glucose intolerance and gluconeogenic upregulation compared with the control group, indicating an essential role of *Tcf7l2* in maintaining glucose homeostasis [83]. Paradoxically, another study found that liver-specific *Tcf7l2* knockout mice with HFD showed significant improvement in glucose tolerance [84]. The discrepancy may be due to the different isoforms of Tcf7l2 that were inhibited. In addition, insulin signaling can also affect bone metabolism. Under insulin resistance, Akt, the downstream of insulin signaling is inhibited, leading to the upregulation of GSK3β, which causes β-catenin degradation and ultimately compromises bone formation effect of Wnt signaling [85]. GSK3β is considered a novel key molecule linking the Wnt pathway and insulin signaling besides sclerostin and DKK1 [85]. Therefore, more basic studies are needed to explore the crosstalk between Wnt signaling and glucose metabolism, and the improvement of glucose metabolism may involve more signaling pathways beyond the Wnt/β-catenin pathway.

## 4. Conclusions and Perspectives

Among the novel biotargeted anti-osteoporotic agents, denosumab has shown potential benefits for glucose metabolism in patients with osteoporosis, aligned with the fact that the pathways they inhibit play a predominantly negative role in glucose homeostasis. Several studies revealed that denosumab reduces HbA1c levels and the risk of diabetes in osteoporotic patients, especially in the elderly and patients with overweight or obesity, possibly via inhibiting NF-κB signalings and elevating GLP-1 levels, which enhances β-cell proliferation and insulin sensitivity. This evidence suggests that denosumab could be a choice for preventing the onset of T2DM when dealing with patients with osteoporosis at high risk of diabetes. However, current clinical studies have several limitations: (1) most of them are observational, and RCTs and cohort studies of long-term denosumab treatment are insufficient; (2) selection bias due to small sample sizes; (3) few studies were conducted on osteoporotic patients with diabetes; (4) incomplete testing of glucose metabolism indicators, including FPG, HbA1c, fasting insulin, HOMA-IR, etc.; and (5) some studies did not control confounders including ADM, diet, exercise, socioeconomic status, and so on. High-quality clinical evidence is urgently required. Additionally, deeper mechanisms of denosumab on glucose metabolism remain to be established.

The Wnt/β-catenin pathway may become another important node linking osteoporosis treatment and glucose metabolism. Although studies have demonstrated that HbA1c and HOMA-IR were positively correlated with sclerostin levels, and that sclerostin suppression ameliorated insulin resistance mediated by ER stress in animal models, the effects of romosuzumab or sclerostin aptamers on glucose metabolism have not yet been confirmed. A reduction in DKK1 levels was identified following ADM, and lower DKK1 expression seemed to correlate with enhanced β-cell survival and proliferation. Nonetheless, the effects of anti-DKK1 antibodies on glucose metabolism urgently warrant exploration.

In summary, it is necessary to investigate more thoroughly the underlying mechanisms by which novel anti-osteoporotic targeted agents influence glucose homeostasis in the future to provide a more appropriate choice of anti-osteoporosis drugs for glycemic control in osteoporotic patients.

## Data Availability

Not applicable.
